# Study on Preparation and Properties of Phosphogypsum-Based Lightweight Thermal Insulation Materials

**DOI:** 10.3390/ma18245476

**Published:** 2025-12-05

**Authors:** Yunpeng Chu, Tianyong Jiang, Han Huang, Gangxin Yi, Binyang Huang

**Affiliations:** 1 School of Civil Engineering and Architecture, Southwest University of Science and Technology, Mianyang 621010, China; chuyunpeng@swust.edu.cn (Y.C.); huanghanswust@163.com (H.H.); yigangxin1@163.com (G.Y.); 13330857917@163.com (B.H.); 2 Shock and Vibration of Engineering Materials and Structures Key Laboratory of Sichuan Province, Mianyang 621010, China

**Keywords:** phosphogypsum, thermal insulation material, mechanical property, microstructure

## Abstract

**Highlights:**

**What are the main findings?**

- The best overall performance was achieved with a 1:4 binder–bead ratio, water–binder ratio of 1.6, 0.1 wt.% HPMC, and 24 wt.% WAE solid content. This formulation yielded low density and thermal conductivity while maintaining adequate strength.
- Adding 8% sulfate aluminate cement improved microstructure densification via interlaced ettringite and gypsum crystals, increasing compressive and flexural strengths by over 20%. Incorporating 0.9% basalt fibers formed a uniform network that dispersed stress and prevented brittle cracking, raising flexural strength by ~18% and improving ductility.
- The combination of 2% paraffin emulsion and spraying 5×-diluted sodium methyl silicate created inner and outer hydrophobic layers. Water absorption decreased by ~23%, and the softening coefficient rose to 0.53, meeting water-resistance requirements.
- SEM and XRD results revealed that cement hydration products (C–S–H gel, ettringite) filled voids among gypsum crystals, while fibers bridged microcracks—jointly enhancing the integrity and stability of the matrix.

**What are the implications of the main findings?**

- The research provides a sustainable method to recycle industrial phosphogypsum waste into high-performance building insulation materials, reducing solid-waste accumulation and related pollution.
- The material’s low thermal conductivity and stable mechanical performance make it a viable alternative to traditional organic insulation products, supporting energy-saving building envelopes.
- The optimized formulation and modification strategy (SAC + fiber + dual waterproof treatment) offer a practical pathway for large-scale production of durable, fire-resistant, and water-resistant inorganic insulation boards.
- The study elucidates the coupling mechanism of cement hydration, gypsum crystallization, and fiber reinforcement in composite matrices—providing a theoretical basis for future design of eco-friendly insulation composites.

**Abstract:**

At present, phosphogypsum, as an industrial by-product, is a solid waste in phosphoric acid production, and its accumulation has caused serious environmental pollution. Furthermore, due to the insufficient insulation properties of traditional wall materials, the issue of a rising proportion of building energy consumption in total social energy consumption has become increasingly pressing. The study investigated vitrified beads as a light aggregate and phosphogypsum, mineral powder, and quicklime as an inorganic composite cementitious system to prepare the phosphogypsum-based lightweight thermal insulation material. The effect mechanism of the initial material ratio on the mechanical properties and micro-morphology of insulation materials was studied by macroscale mechanical property testing, X-ray diffraction, and scanning electron microscopy. Meanwhile, in order to meet the performance indexes specified in relevant standards, insulation materials were modified by adding sulfate aluminate cement, basalt fibers, and a waterproof agent to improve the strength, toughness, and water resistance. Based on the single-factor experimental design, the optimal dosage of various admixtures was obtained. The results indicated that the optimal properties of the sample were achieved when the binder–bead ratio was 1:4, the water–binder ratio was 1.6, the dosage of hydroxypropyl methylcellulose was 0.1%, and the solid content of waterborne acrylic emulsion was 24%. The optimal dosages of cement and fibers were 8% and 0.9%, respectively. The cement hydration products and gypsum crystals lapped through each other, filling the pores in the matrix and increasing the strength of the sample. In addition, the fibers could form a disordered network structure inside the matrix, disperse external force, weaken the stress concentration at the tip of internal cracks, and significantly improve the toughness of the modified sample. By incorporating 2.0% paraffin emulsion in the mortar and spraying 5 dilutions of sodium methyl silicate on the external surface, dense protective layers were formed both inside and outside the modified sample. The water absorption rate reduced from 30.27% to 23.30%, and the water resistance was increased to satisfy the specified requirement for the insulation material.

## 1. Introduction

Insufficient insulation properties of buildings have caused substantial energy loss [1,2]. Existing research demonstrates that adhering the insulation material to the external wall of buildings is one of the most effective methods to improve building energy efficiency [3,4]. Common insulation materials used in walls are mainly divided into three types: metallic insulation materials, organic insulation materials, and inorganic insulation materials. Metallic materials exhibit outstanding thermodynamic stability and resist heat transfer, with variable environmental conditions, but the application is limited due to low output and high cost [5,6]. Organic insulation materials occupy the majority of the insulation material market due to characteristics such as lightweight, good thermal insulation, and water resistance. However, organic materials also expose shortcomings in engineering applications, such as easy combustion and aging during use [7,8]. In terms of fire safety and energy savings, inorganic insulation materials offer enormous property advantages over the other two types of material [9,10]. Currently, phosphogypsum, as a by-product of industrial production, faces the challenge of having a limited range of application fields and a low utilization rate. If the advantages of phosphogypsum, including lightweight, fire resistance, high plasticity, and low thermal conductivity, can be fully utilized to produce the insulation material, this method not only reduces building energy consumption but also relieves the difficulties of the gypsum industry [11,12]. Converting this industrial by-product into building insulation material not only mitigates the environmental burden associated with waste accumulation but also reduces the carbon footprint of the final product by partially replacing energy-intensive binders such as cement. This study aims to alleviate the large-scale accumulation and underutilization of industrial phosphogypsum by converting it into a high-value lightweight thermal insulation material. Developing inorganic insulating cementitious materials is crucial for improving building energy efficiency while providing fire resistance, durability, and environmental sustainability, thereby offering a greener alternative to organic insulation products.

Existing research and development of wall insulation materials focus on low-density and superior thermal insulation properties [13]. In particular, in the production of phosphogypsum-based lightweight thermal insulation materials, reducing the density of materials has been regarded as an important technical trend [14]. Foaming technology and lightweight aggregates, as two main methods, are usually used to achieve the purpose. Both physical foaming and chemical foaming can generate closed pores inside the material matrix, improving the insulation properties, but the forming quality of pores is difficult to control. The commonly used lightweight aggregates include vitrified beads, expanded perlite, and expanded polystyrene (EPS) [15,16,17]. The composite quality of the lightweight aggregate and the cementing material plays a crucial role in the properties of insulation materials [18]. Gypsum belongs to gas-hardening cementitious material, which has poor water resistance and is prone to corrosion when exposed to water. It has been reported that the addition of admixtures to the mortar mixture to optimize the pore structure can overcome the shortcomings of the cementitious material [19]. Magdaléna et al. [20] studied a new gypsum composite material that was prepared with a flue gas desulfurization binder and three different types of fine fillers. The results indicated that the type and surface quality of the aggregate had a significant influence on the shape and size of gypsum crystals. As the roughness of the particulate surfaces increased, there was a concomitant enhancement in the mortar’s mechanical properties. Celik et al. [21] developed thermal insulation mortar using vitrified beads, which exhibited excellent anti-aging properties and high temperature resistance. However, its water absorption and thermal conductivity were high. The surface of the vitrified beads required treatment prior to application. Zhao et al. [22] studied the influence of the sodium methyl silicate dosage on the water resistance of the gypsum sample and elaborated the mechanism of the waterproof agent from the microstructure of the sample. Xie et al. [23] incorporated basalt fibers of different lengths into phosphogypsum in different proportions to prepare matrix composites. The results showed that the fibers, at a dosage of 1.31% and a length of 6 mm, not only apparently improved the strength and toughness of the mixture but also reduced the porosity and water absorption. Although gypsum-based insulation materials have shown great advantages and potential in many aspects, there are still problems of insufficient insulation property, high density, and construction difficulty in practical application. Additionally, the building material market lacks specific foaming agents for gypsum products, so cement foaming agents are commonly used as substitutes. This situation reflects the imperfections in the preparation process of gypsum lightweight insulation materials, which warrant extensive experimental research [24,25,26,27].

To broaden the application prospects of phosphogypsum, reduce the burden of accumulation, and realize resource application, phosphogypsum-based lightweight thermal insulation materials were prepared in this study. The material was composed of vitrified beads as the lightweight aggregate and an inorganic composite cementitious material composed of phosphogypsum, mineral powder, and quicklime. In this system, quicklime hydrates to produce Ca(OH)_2_, which increases the alkalinity and facilitates the activation of phosphogypsum and mineral powder, thereby promoting the formation of cementitious reaction products. Furthermore, the basalt fiber and sulfate aluminate cement (SAC) were incorporated to improve mechanical strength, and water-reducing agent, retarder, and hydroxypropyl methylcellulose (HPMC) were added to strengthen the construction performance. Through a series of single-factor experiments, the effects of different admixture dosages on the properties of the samples were analyzed in terms of mechanical properties and micro-morphology, which provided data to support research into the preparation of insulation materials.

## 2. Materials and Methods

### 2.1. Raw Material

**Figure 1:**
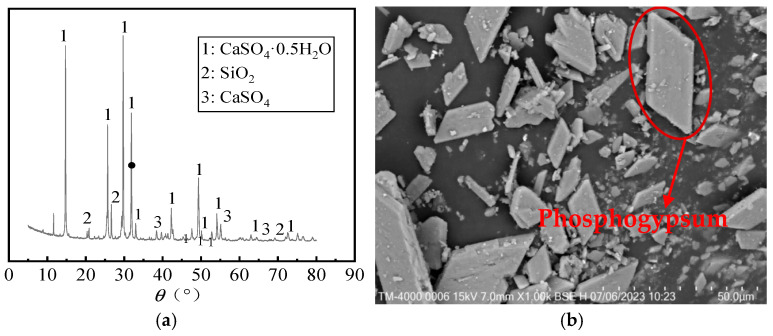
The detection images of phosphogypsum: (**a**) XRD pattern; (**b**) SEM image.

**Figure 2:**
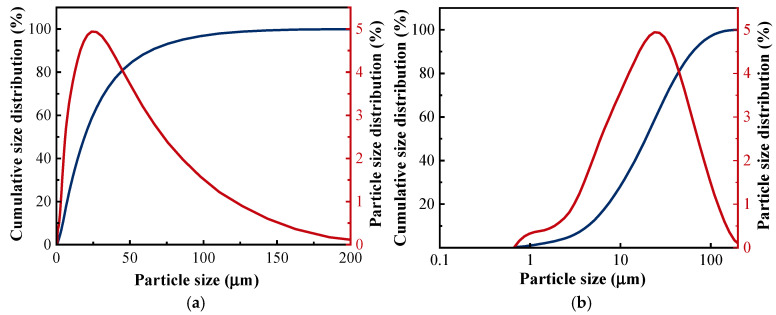
Particle size distribution of raw materials: (**a**) phosphogypsum; (**b**) quicklime.

The phosphogypsum provided by Hongda Company (Chengdu, China) is a grayish-white powder with a 12% water content. The X-ray diffraction (XRD) pattern and scanning electron microscopy (SEM) are shown in Figure 1a and Figure 1b, respectively. The main mineral composition of phosphogypsum is calcium sulfate dihydrate. The particle size distribution ranges from 0.767 to 163 µm, with an average particle size of 36.47 µm. The micro-morphology shows a flake-like feature. The mineral powder produced by Casting Materials Company (Beijing, China) is classified as S95 grade, exhibiting a grayish-white powder. The X-ray fluorescence spectrometer (XPF) is used to analyze the chemical components of phosphogypsum and mineral powder, as shown in Table 1. The particle size distribution of phosphogypsum and mineral powder is shown in Figure 2. The fluidity, water resistance, and reactivity of phosphogypsum can be improved by adding mineral powder. Quicklime produced by the National Pharmaceutical Group is a white powder solid material. The fluoride ions contained in phosphogypsum can be modified by quicklime to form the insoluble precipitate calcium fluoride (CaF2), effectively reducing the content of soluble fluoride ions. Its reactivity was evaluated according to standard GB/T 5762-2024 [28]. The measured reactivity R60 was 152 °C, indicating a medium–high reactivity level that ensures sufficient participation in the fluorine-fixation reaction forming CaF2. Sulfate aluminate cement (SAC) was obtained from Zhonglian Cement Factory, and the chemical composition is shown in Table 1. Vitrified beads were crafted from acidic resinous rock through a high-temperature process in a vitrified furnace. The surface was covered with a vitrified hard shell, and the interior exhibited a spherical honeycomb structure. These beads feature good physical and chemical stability, thermal insulation, and fire resistance. The physical properties of vitrified beads are shown in Table 2. The basalt fiber, provided by Changsha Huixiang Company (Changsha, China), can decrease cracks during the drying shrinkage of mortar. HPMC was obtained from Chenqi Chemical Technology Company (Shanghai, China); it is a white, powdery, solid particle with a water retention function. The waterproofing agents include paraffin emulsion and sodium methyl silicate (SMS), both of which are produced by Yousuo Chemical Technology Company (Heze, China). The solid content of the two waterproofing agents is 30%. Waterborne acrylic emulsion (WAE) was made of pure acrylic monomer copolymerization emulsion, which has good water resistance and alkaline resistance.

### 2.2. Ratio and Preparation of Insulation Materials

Composite cementitious materials were made from phosphogypsum, quicklime, and mineral powder by weight ratio. According to the previous single-factor experimental results, only the mineral powder was internally doped, with a mass fraction of 20%. The other admixtures were externally doped, including 0.1% CQ-SHJ09 (protein-based retarder), 2% quicklime (alkali activator), and 0.15% polycarboxylate superplasticizer. The mineral powder promoted secondary hydration and refined the pore structure of the phosphogypsum-based binder. The protein-based retarder delayed the setting time, preventing premature stiffening of the slurry. Quicklime reacts with soluble fluoride ions to form insoluble CaF2, reduces the negative effects of impurities, and provides an alkaline environment that enhances binder reactivity. The polycarboxylate superplasticizer improves particle dispersion and increases fluidity, enabling better mixing and lower water demand. These additives jointly enhance the workability, stability, and hydration behavior of the composite cementitious binder. To prepare the insulation material, different dosages of vitrified beads, HPMC, and WAE need to be added to the cementitious material. The initial material ratio is shown in Table 3. Furthermore, to enhance the mechanical properties and water resistance of the insulation material, SAC, basalt fibers, paraffin emulsions, and SMS were incorporated into the composition. The modified material ratio is also shown in Table 3. It should be noted that the fluidity in this study was measured on the fresh mixture immediately after mixing, whereas all other performance tests were conducted on hardened samples.

**Figure 3:**
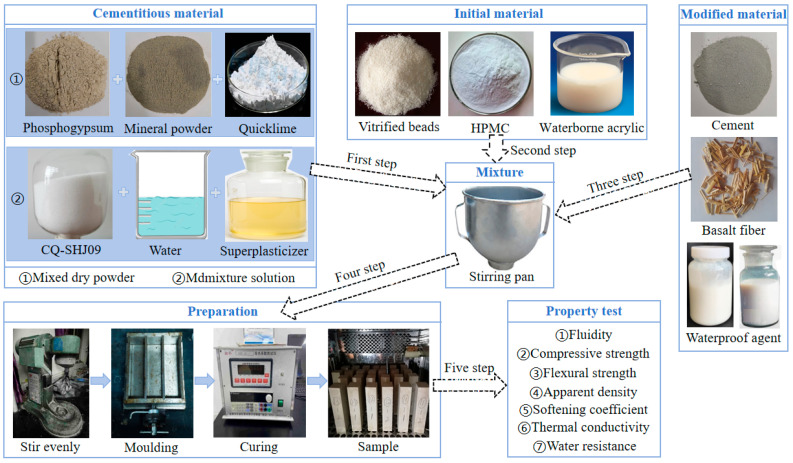
Preparation of phosphogypsum-based lightweight thermal insulation material.

The preparation process for the insulation material is described in Figure 3. (1) Mixed dry powder was prepared by low-speed agitation of phosphogypsum, mineral powder, and quicklime for a duration of 30 s. During dry blending of phosphogypsum, mineral powder, and quicklime, the solid particles were redistributed, achieving closer contact. Mineral powder fills the voids between phosphogypsum particles, improving initial packing density. Quicklime partially interacts with residual moisture adsorbed on particle surfaces, forming small amounts of Ca(OH)_2_. This behavior corresponds to the moisture-induced surface pre-hydration of CaO reported in gypsum-based systems, which begins stabilizing soluble fluorides through the formation of CaF2. Because only limited surface-bound water is available, this stage does not trigger bulk hydration, but it allows the powders to form a more uniform and compact initial microstructure that determines later reaction interfaces. (2) Retarder and superplasticizer were added into water and stirred for 60 s to obtain the admixture solution. When the retarder and superplasticizer dissolve in water, they interact with solid surfaces after mixing with the dry powder. The retarder adsorbs onto gypsum surfaces, delaying early CaSO_4_·0.5H_2_O to CaSO_4_·2H_2_O hydration. The superplasticizer disperses the cementitious particles and reduces flocculation, improving wetting and fluidity. The admixture solution also allows HPMC, fibers, and paraffin emulsion to pre-disperse, forming more uniform interfaces before hydration begins. This stage creates a lubricated and better-separated particle system, which promotes a more homogeneous microstructure during setting. (3) The raw materials for the second and third steps need to be separately incorporated into the distinct mixtures prepared in the first step. The vitrified beads and cement were added to the mixed dry powder. HPM, fibers, and paraffin emulsion were added to the admixture solution. Subsequently, the mixed dry powder and admixture solution were mixed in the design proportion to produce the gypsum mixture. Once the admixture solution and dry mixture were combined, significant microstructural development occurred. Gypsum hydration produces interlocking CaSO_4_·2H_2_O crystals that form the primary framework. SAC hydrates to generate ettringite and C-S-H gel, which fill pores and strengthen the matrix. Basalt fibers become embedded in the developing matrix and act as micro-bridges to resist crack initiation and propagation. HPMC retains water and creates micro-air pores that improve workability and pore stability. Paraffin emulsion disperses as droplets and later forms hydrophobic domains on crystal surfaces. WAE forms a thin polymer film that enhances interface compatibility with vitrified beads and reduces surface absorption. (4) The mixture was stirred at low speed for 60 s to ensure uniformity. It was then cast into the standard sample mold. To prevent the formation of pores inside the sample, the mixture had to be repeatedly oscillated on a mortar shaking table and then meticulously leveled with a trowel. After standing for 18 h under standard laboratory conditions, the samples were transferred to a steam curing chamber at 17 °C for 21 h. This curing protocol reflected the actual production conditions of prefabricated phosphogypsum-based insulation materials. The mechanical and water-resistance tests conducted at later ages showed stable trends, indicating that the results are not overly sensitive to this curing regime. The initial 18 h rest under laboratory conditions allows the fresh mixture to complete early gypsum hydration and establish a preliminary crystal framework. During this period, CaSO_4_·0.5H_2_O gradually transforms into interlocking CaSO_4_·2H_2_O crystals, and the slurry gains sufficient early strength to maintain dimensional stability before steam curing. The subsequent 21 h steam curing at 17 °C accelerates the hydration of SAC, promoting the formation of ettringite and C-S-H gel that fills internal pores and further densifies the matrix. The mild temperature prevents thermal cracking while still enhancing moisture availability for sustained hydration. This curing stage also improves the bonding between gypsum crystals, WAE, basalt fibers, and vitrified beads, thereby enhancing both mechanical strength and water resistance. After curing, the samples were demolded and placed for the corresponding age.

The parameter screening in this study follows a stepwise arrangement in which the main variables are examined individually. Each parameter affects a different aspect of the material system, including packing, water retention, rheology, and interfacial bonding. Within the studied ranges, these mechanisms remain largely independent, allowing single-factor adjustment to provide a clear basis for determining effective parameter ranges before the composite mixture is evaluated.

### 2.3. Performance Testing and Characterization of the Thermal Insulation Material

Phosphogypsum slurry was prepared according to the standard GB/T 17669.4-1999 [31], and both the water consumption and setting time of the standard consistency were measured. Prism-shaped samples, with dimensions of 40 mm × 40 mm × 160 mm, were prepared and cured for the corresponding age to test the compressive and flexural strengths. The fluidity of the insulation mortar was assessed by referring to the Chinese Code [32], using the standard consistency tester according to the Redhead method. Fluidity is defined as the spread diameter of the fresh mortar measured after lifting the standard consistency mold, which reflects the flow ability of the mixture under its self-weight. Water resistance was characterized by the softening coefficient and water absorption rate. According to the standard GB/T 50082-2009 [33], the softening coefficient of the sample is the ratio of the ultimate compressive strength after water saturation (R1) to that in the dry state (R2). R1 was tested after the sample had been immersed in clean water for 24 h, and the surface moisture was then wiped away. R2 was tested after the sample had been dried to a constant weight in a dry environment at (40 ± 2) °C. Furthermore, according to the standard GB/T 9775-2008 [34], the water absorption was determined by measuring the amount of water absorbed per unit volume of samples after 2 h and 24 h. The thermal conductivity was measured using a DRE-2C type thermal conductivity tester [29]. All measurements were conducted on three parallel specimens, and the recorded results represent the mean values. The variability among the three specimens was small, and preliminary calculations indicated that the coefficient of variation was within an acceptable range for all tests. The microstructure of the hardened samples was observed using a scanning electron microscope operated in backscattered-electron mode at an accelerating voltage of 15 kV. The specimens were fractured to expose fresh internal surfaces, dried at 40 °C, mounted on conductive stubs, and sputter-coated with a thin gold layer prior to imaging.

## 3. Results and Analysis

### 3.1. Study on the Initial Material Ratio of the Thermal Insulation Material

#### 3.1.1. Effect of Vitrified Beads Dosage on the Material Properties

**Figure 4:**
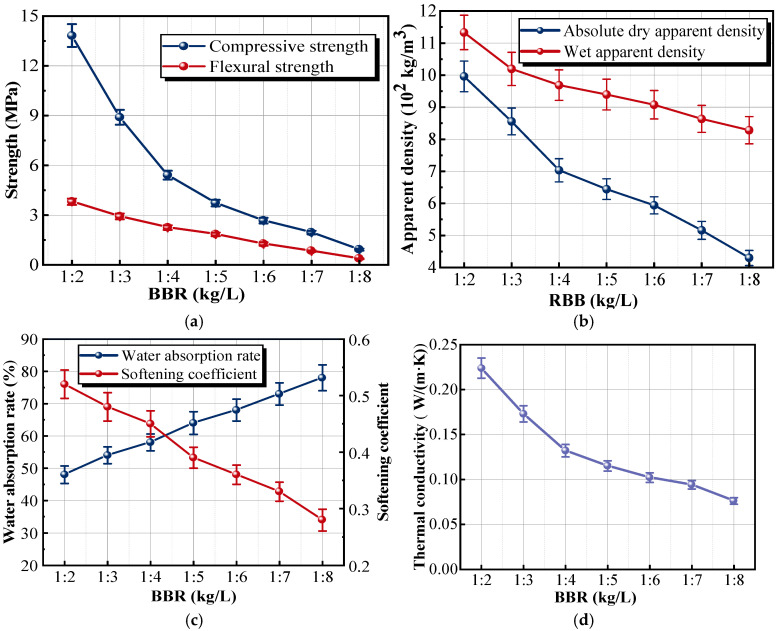
Effects of different BBR on the properties of samples: (**a**) strength; (**b**) apparent density; (**c**) water absorption rate and softening coefficient; (**d**) thermal conductivity.

The series of experiments investigates how the ratio of the cementitious material mass (kg) to the vitrified beads volume (L), known as the binder–bead ratio (BBR), affects the properties of the insulation material. The results are shown in Figure 4. As the dosage of vitrified beads increased, the content of cementitious material within a unit volume of the sample decreased accordingly. Because the inherent strength of vitrified beads was low and contributed little to the strength of insulation materials, the curve exhibited a downward trend, as shown in Figure 4a. When the dosage of vitrified beads was 2 L, the compressive and flexural strengths of the sample were the highest, which were 13.83 and 3.81 MPa, respectively. Compared with the strengths of the sample with 1:2 BBR, the compressive and flexural strengths of the sample with 1:5 BBR were reduced by 73.03% and 40.95%, respectively. The main reason was that when the BBR of the sample exceeded 1:4, the hydration products generated in the insulation material could not completely wrap the vitrified beads. The internal structure of the material matrix became relatively loose, and the strength of the sample fell below the minimum mechanical requirements specified in GB/T 9775-2008 [34], which stipulate a compressive strength of not less than 5.0 MPa and a flexural strength of not less than 2.5 MPa.

With the increase in the vitrified beads dosage, the apparent density and softening coefficient of the sample decreased, while the water absorption rate gradually increased, as shown in Figure 4b,c. Specifically, the absolute dry and wet apparent densities of the sample with 1:4 BBR were 968.8 and 703.1 kg/m^3^, respectively. The vitrified beads had a porous structure with a vitrified and closed surface. As the dosage of vitrified beads continued to increase, the workability of the insulation mortar deteriorated, resulting in a substantial increase in water consumption for preparing insulation materials. In addition, the reduction in the cementitious material content decreased the encapsulation and sealing ability of the mortar to aggregate openings and increased the porosity between aggregates inside the matrix. Therefore, the apparent density and softening coefficient of the sample decreased, and the water absorption per unit volume increased.

Figure 4d provides the trend of the thermal conductivity, which decreases with the increasing dosage of vitrified beads. Compared with the sample with 1:2 BBR, the thermal conductivity of the sample with 1:4 BBR dropped from 0.224 to 0.132 W/(m·K) with a reduction of 41.07%. The insulation property of the material was improved. The vitrified beads in samples, characterized by plenty of sealed pores, enhanced the thermal resistance. Consequently, the higher dosage of vitrified beads in insulation material was directly correlated with a reduction in thermal conductivity. However, when the BBR in the sample exceeded 1:4, the decrease rate of the thermal conductivity slowed down. The reason was that an excessively high dosage of vitrified beads could increase the frequency of collision with the agitation blades and mixing vessel during the mixing process, resulting in a higher breakage rate. The results indicated that the optimal BBR was 1:4.

#### 3.1.2. Effect of the HPMC Dosage on the Material Properties

**Figure 5:**
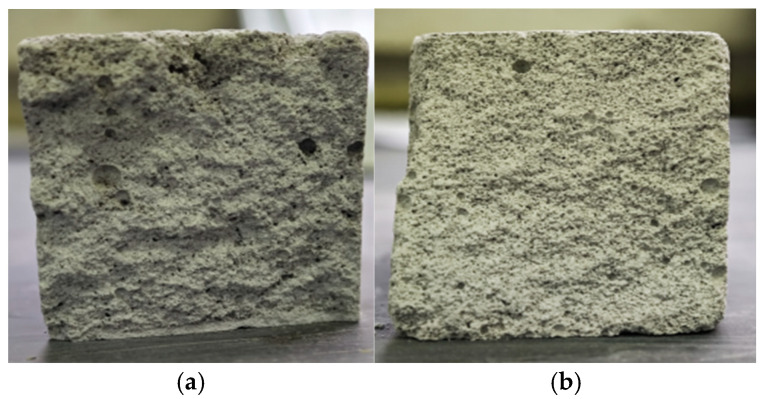
Photos of the sample section: (**a**) the reference sample; (**b**) the sample mixed with HPMC.

As shown in Figure 5a, the sample without HPMC exhibits extremely poor water retention, which is associated with significant bleeding. The sectional morphology features a rough texture, and the insulation mortar only wraps part of the aggregate. Figure 5b depicts the sample mixed with HPMC, in which the water retention is improved. Furthermore, there is no observable loss of the insulation mortar, the bleeding phenomenon in the mortar is almost eliminated, and the sample’s surface displays a high degree of planarity.

**Figure 6:**
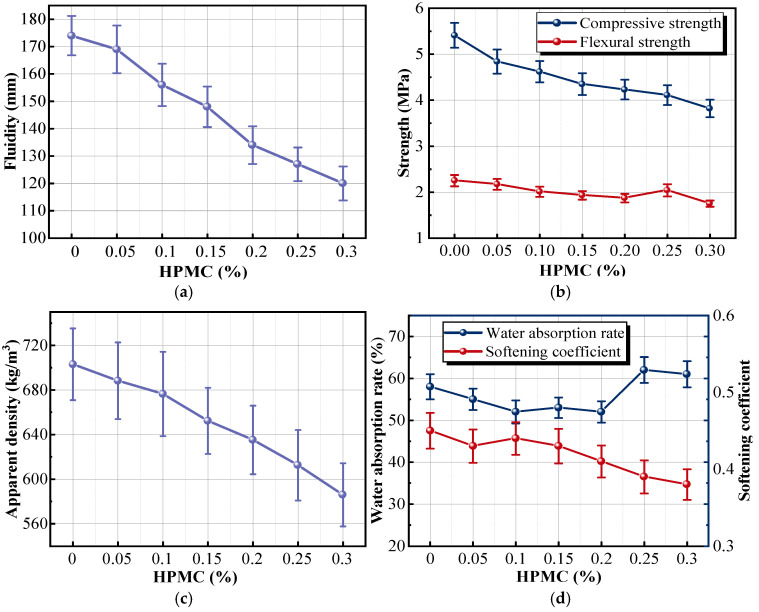
Effects of different HPMC dosages on the properties of samples: (**a**) fluidity; (**b**) strength; (**c**) apparent density; (**d**) water absorption rate and softening coefficient.

With the increase in HPMC dosage, the water retention of the insulation mortar was enhanced, and its apparent viscosity increased, as reflected by the reduced fluidity, as shown in Figure 6a. The intermolecular van der Waals forces in HPMC conferred exceptional water retention properties to the mortar, which was essential for strengthening its stability. In this study, viscosity was not measured directly; instead, changes in apparent viscosity were inferred from the fluidity (spread diameter) measured according to standard GB/T 2419-2005 [35]. However, when the HPMC dosage exceeded the critical level of 0.15%, these benefits were significantly restricted. This is because excessive HPMC rapidly binds free water into a gel-like network, which accelerates early stiffening and shortens the workable construction time. The accelerated setting rate and decreased fluidity posed greater challenges in construction. The mixing process must be completed within a short time, severely increasing the difficulty of construction operations.

Figure 6b,c show that the strength and apparent density of the sample were negatively correlated with the HPMC dosage. The flexural and compressive strengths of the sample with 0.1% HPMC were 4.62 and 2.01 MPa, which were 15.57% and 16.10% lower than those of the reference sample, respectively. When the dosage exceeded 0.20%, the flexural strength curve had an upward trend, but the peak point was still 9.33% lower than that of the reference sample. The principal cause was attributed to the thickening effect of HPMC. As reported in previous studies, the methoxyl (CH_3_O–) and hydroxypropoxyl (–OCH_2_CHOHCH_3_) groups distributed along the cellulose ether backbone enhance the ability of hydroxyl and ether oxygen atoms to form hydrogen bonds with water, thereby converting free water into bound water [36]. This mechanism is consistent with the observed reduction in fluidity (Figure 6a) and the increased porosity reflected by the decreased apparent density (Figure 6c). The insulation mortar increased the initial moisture content. As the mortar gradually dried and molded, the moisture evaporated, leaving lots of tiny pores in the sample. These pores not only disrupted the continuity of the mortar matrix but also severely affected the stability of the internal structure, consequently weakening the mechanical strength of the sample. Furthermore, HPMC contained alkyl groups (CH_3_-), which could reduce the surface energy of aqueous solutions. The toughness of the modified mortar bubble film was higher than that of the blank mortar bubble film, which promoted an increase in gas content of the mortar [37]. The increase in porosity was conducive to decreasing the apparent density of the sample.

As shown in Figure 6d, the water absorption rate of the sample with 0.1% HPMC reduced from 58% to 52%, compared with the reference sample. When the dosage reached 0.25%, the water absorption rate was the highest. The softening coefficient of the sample with 0.1% HPMC reduced from 0.47 to 0.43 with a reduction of 8.51%, compared with the reference sample. When the dosage exceeded 0.1%, the softening coefficient curve dropped sharply. The excessive addition of HPMC could stimulate serious foaming in the mortar, and the fusion and penetration of microbubbles increased the porosity. Meanwhile, excessive dosage also impacted both the construction efficiency and quality of the sample, which manifested in poor workability, high viscosity, and difficulty in leveling and shaping. Therefore, the optimal dosage of HPMC was 0.1%.

#### 3.1.3. Effect of the Water–Binder Ratio on the Material Properties

In this group’s experiment, the dosage of the other components was kept constant to study the effect of the water–binder ratio (WBR) on the properties of the insulation material.

**Figure 7:**
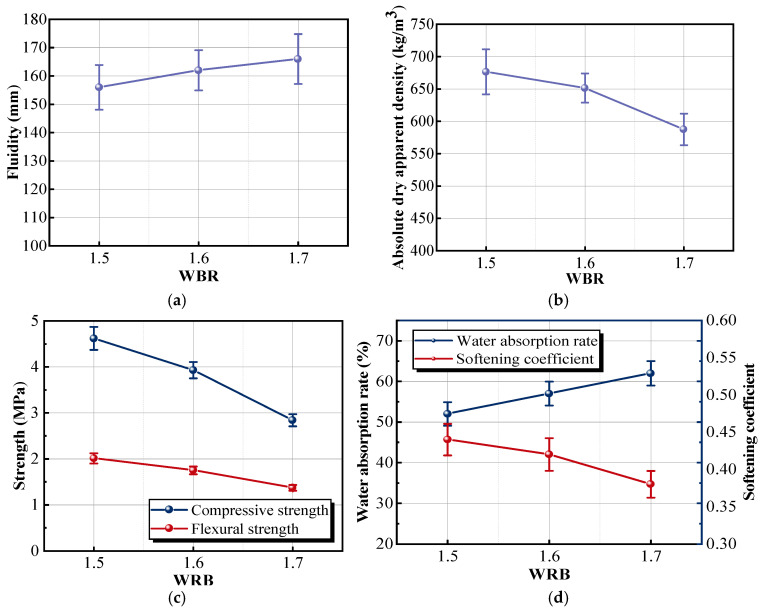
Effects of different WBR dosages on the properties of samples: (**a**) fluidity; (**b**) absolute dry apparent density; (**c**) strength; (**d**) water absorption rate and softening coefficient.

With the increase in the WBR, the fluidity curve of the insulation mortar exhibits an upward trend, whereas the apparent density curve gradually declines, as shown in Figure 7a,b. When the WBR of the mortar was kept at 1.5, the fluidity only reached 156 mm. At this low WBR, the mortar exhibited a high resistance to deformation, corresponding to a high yield stress or apparent viscosity, which in turn resulted in poor fluidity. This reduced flowability increased the difficulty of mixing the mortar and easily caused uneven distribution of components in the mortar. Compared with the sample with a WBR of 1.5, the fluidity of the sample with a WBR of 1.7 was increased by 6.41%, and the apparent density was decreased by 13.16%. Cementitious material was more easily dispersed in high-water-content mortar, and the bubble was more evenly wrapped. This improvement in fluidity with increasing WBR can be explained by physicochemical mechanisms. A higher WBR increases the amount of free water, which reduces particle–particle friction and provides better lubrication between solid particles. The diluted binder concentration weakens the flocculated gel network formed during early hydration, decreases the yield stress of the slurry, and reduces viscosity. As a result, the mortar becomes more deformable under shear, and its flowability increases. The changes of the strength curve, water absorption rate curve, and softening coefficient curve are shown in Figure 7c,d. With the increase in the WBR, the strength and water absorption rate of the sample decreased, and the softening coefficient gradually increased. Compared with the sample with a WBR of 1.5, the compressive and flexural strengths of the sample with a WBR of 1.7 were 2.81 and 1.30 MPa, with a reduction of 39.10% and 35.02%; the softening coefficient reduced from 0.44 to 0.38, and the water absorption rate was increased by 18.67%. Although increasing the moisture content raised the dissolution space of cementitious material, the proportion of the cementitious material was smaller. During the curing of the sample, only a little water participated in the hydration reaction of the mixture. More water was dissipated by evaporating, which formed many pores in the matrix. Loose internal structure reduced the mechanical properties and improved the water absorption rate. A higher WBR results in increased water absorption due to microstructural changes during curing. Only a small portion of the mixing water participates in hydration, while the excess water evaporates and leaves behind a large number of capillary and interconnected pores. These pores enlarge the pathways for water ingress and increase the overall porosity of the matrix. The resulting discontinuity of the mortar skeleton weakens mechanical integrity and makes the material more vulnerable to moisture-induced degradation, thereby adversely affecting long-term durability. The above results showed that the optimal WBR of the mortar was 1.6.

#### 3.1.4. Effect of Surface Treatment of Vitrified Beads on Properties of Thermal Insulation Materials

**Figure 8:**
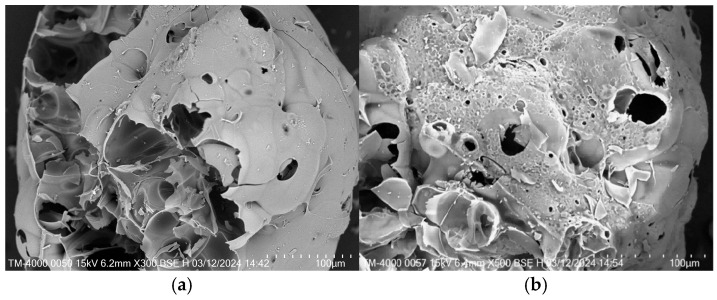
Micrographic comparison of vitrified beads: (**a**) original vitrified beads; (**b**) modified vitrified beads.

Vitrified beads are a great lightweight aggregate with a smooth surface and closed pore structure. However, Figure 8a shows that the bead surface is easily damaged. The mixing process changed the original closed structure to one with more pores, reducing the thermal insulation property. As shown in Figure 8b, the bead surface modified by waterborne acrylic emulsion (WAE) was covered by a layer of polymer film. Even after agitation, the surface morphology of the aggregate remained intact, and the coating layer was hardly damaged. Notably, the preparation process for the modified vitrified beads involved the following steps: (1) The initial WAE was mixed with water in a certain proportion to obtain a uniform emulsion with solid contents of 8%, 16%, 24%, 32%, and 40%, respectively. (2) An amount of WAE equivalent to 20% of the mass of the vitrified beads was weighed. (3) The prepared WAE was slowly poured into the vitrified beads and thoroughly mixed to ensure that the emulsion could be effectively attached to the surface of the vitrified beads. Table 4 indicates that the packing density and cylinder compressive strength of the modified beads were positively correlated with WAE dosage. The strength of the beads with a WAE solid content of 40% increased from 115 to 303 kPa with an increase of 163.47%, compared with the blank bead. Considering the preparation cost and modification efficacy, the optimal solid content of WAE was 24%. The packing density of the vitrified beads was 110.2 kg/m^3^, the water absorption rate was reduced by 34.2%, the cylinder compressive strength increased by 89.57%, and the floating rate was more than 90%, all of which met the physical property requirements of lightweight aggregates specified in the Chinese code [30].

**Figure 9:**
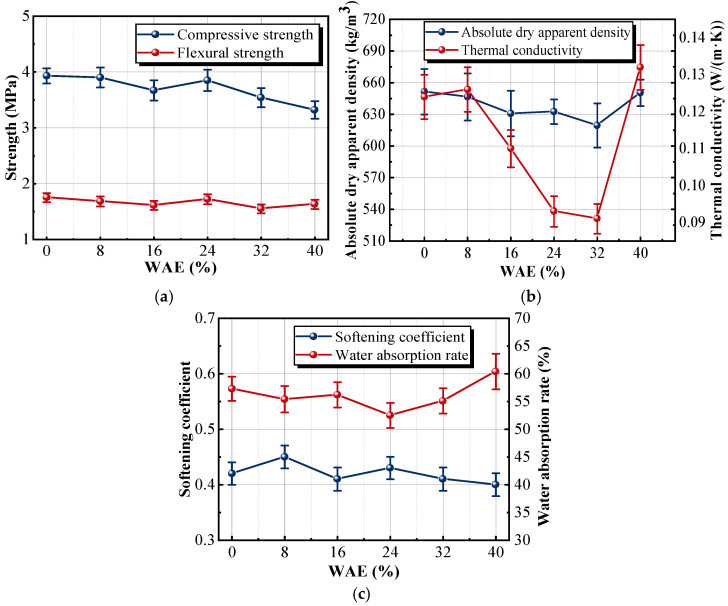
Effects of different WAE dosages on the properties of samples: (**a**) strength; (**b**) absolute dry apparent density and thermal conductivity; (**c**) water absorption rate and softening coefficient.

As shown in Figure 9a, the strength of the modified sample is smaller than that of the reference sample. The strength curves of the sample with 24% WAE solid content rebounded due to an 89.57% increase in the cylinder compressive strength of the vitrified beads, but the compressive and flexural strengths of the sample were still lower than those of the unmodified group. The weak part of the insulation material was the bonding interface between the cementitious material and the lightweight aggregate. The strength of the sample was determined by the thickness of the cementitious material wrapping the aggregate [38]. Although WAE could improve the cylinder compressive strength of the vitrified beads to reduce the breakage rate of the aggregate during the mixing process, it also increased the volume of the aggregate in the material. The thinning of the coating thickness of the cementitious material weakened the bonding interface between the aggregate and the cementitious material, making the sample prone to cracking.

While the WAE treatment increased the cylinder compressive strength of the vitrified beads through the formation of a reinforcing polymer film, this localized enhancement did not translate into a higher overall compressive strength of the composite. The polymer film has much lower stiffness than the mineral-based skeleton of the cementitious matrix, resulting in inefficient load transfer between the beads and the binder. In addition, the polymer-coated regions disrupt the continuity of the hardened matrix and weaken the mechanical interlock at the bead–binder interface. Consequently, even though individual beads become more resistant to local deformation, the global compressive strength of the composite remains lower than that of the unmodified group. The interfacial transition zone between the cementitious binder and the vitrified beads forms the weak region of the composite because the smooth bead surface limits bonding. The thickness of the binder coating around the beads governs the effective load-transfer area. When WAE modification increases the effective bead volume, the binder layer becomes thinner, reducing the load-bearing cross-section and causing stress concentration. As a result, the overall strength decreases even though the local strength of the beads is improved.

As the WAE solid content increases from 0 to 32%, the apparent density curve and thermal conductivity curve show a decreasing trend in Figure 9b. The sample with 32% WAE solid content had the lowest apparent density, which was 4.9% lower than that of the reference sample (651.4kg/m^3^). The reduction in apparent density is attributed to the increased proportion of aggregates within the unit volume of the insulation material. Concurrently, the thermal conductivity dropped to 0.093, and the insulation property of the material was enhanced. As shown in Figure 9c, when the WAE solid content exceeds 24%, the water absorption rate curve of the sample shows an upward trend, whereas the softening coefficient curve shows a downward trend. When the WAE dosage becomes excessive, the polymer fraction in the composite increases, and the hydrophilic functional groups in WAE enhance the material’s affinity for water, resulting in higher water absorption. At the same time, an overly thick polymer film forms around the vitrified beads, which interferes with the normal hydration of the cementitious matrix and creates a weaker and more porous interfacial transition zone. Under wet conditions, this weakened interface deteriorates more rapidly, leading to a lower softening coefficient. The water absorption rate and thermal conductivity of the sample with 40% WAE solid content were the highest, which were 7.5% and 20% higher than those of the sample with 24% WAE solid content, respectively. The sample with 40% WAE solid content had the lowest softening coefficient, at only 0.4. The molecular structure of WAE contained numerous hydrogen bonds, which could interact with water molecules. Upon the incorporation of WAE into the insulation material, the high-water-absorption property was imparted to the sample. Furthermore, an appropriate amount of WAE could form a uniform film on the surface of vitrified beads, effectively reducing the thermal conductivity and blocking the direct heat transfer path. However, when the solid content was excessive, the polymer latex film exacerbated the thermal bridge effect, resulting in a decrease in thermal resistance. This reduction in thermal conductivity is related to the microstructural effects of the WAE polymer film. A higher solid content forms a more continuous coating on the surface of the vitrified beads, helping preserve their closed-pore structure and reducing solid–solid contact between the beads and the surrounding matrix. This increases the proportion of air-filled pores and disrupts continuous heat-transfer pathways, thereby lowering bulk density and improving insulation performance.

A higher dosage of vitrified beads effectively decreases thermal conductivity by lowering density and increasing the proportion of closed pores. HPMC contributes to improved pore stability and slightly enhances insulation performance when used at an appropriate level, but excessive amounts may introduce interconnected pores and increase thermal conductivity. In contrast, increasing WAE dosage tends to raise thermal conductivity because the polymer film formed during curing has higher thermal conductivity than air, although it simultaneously enhances water resistance. These combined effects demonstrate that optimized dosages of the three components are essential for balancing insulation performance and mechanical durability.

### 3.2. Study on the Modified Material Ratio of the Thermal Insulation Material

The insulation material prepared using the initial material ratio exhibits lower thermal conductivity. However, this advantage is diminished due to insufficient mechanical properties and water resistance, posing a challenge for engineering applications. To address these problems, the effects of SAC, basalt fibers, and waterproof agents on strengthening the mechanical properties and water resistance of the insulation material were investigated through single-factor experiments.

#### 3.2.1. Effect of SAC Dosage on the Material Properties

**Figure 10:**
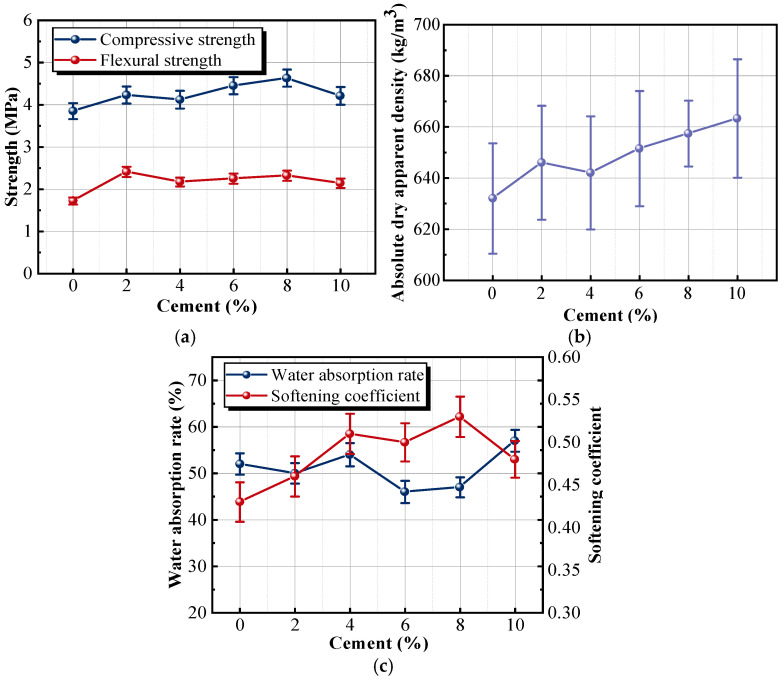
Effects of different WAE dosages on the properties of samples: (**a**) strength; (**b**) absolute dry apparent density; (**c**) water absorption rate and softening coefficient.

The curves of flexural strength and compressive strength show normal distribution in Figure 10a. The compressive and flexural strengths of the sample with 8% cement were 4.63 and 2.31 MPa, which showed an increasing trend of 20.3% and 34.3%compared with the reference sample, respectively. Although the absolute strength values remained moderate, the differences between the 8% and 10% cement groups exceeded the measured variability among the three parallel specimens, indicating that the observed trend is robust within the scope of this study. However, when the dosage reached 10%, the compressive and flexural strengths decreased to 4.21 and 2.14 MPa, respectively. The hydration rate and mechanism of cement and composite cementitious materials were different. Phosphogypsum was hydrated from calcium sulfate hemihydrate (CaSO_4_·0.5H_2_O) to calcium sulfate dehydrate (CaSO_4_·2H_2_O). The primary components of cement, including tricalcium aluminate (Ca_3_Al_2_O_6_), dicalcium silicate (Ca_2_SiO_4_), and tricalcium silicate (Ca_3_SiO_5_), generated various hydration products during the reaction process, which played a crucial role in the hardening and strengthening of the sample. Ca_3_Al_2_O_6_ was readily hydrated in supersaturated gypsum slurry to form needle-like ettringite (Aft). Meanwhile, Ca_2_SiO_4_ reacted with Ca_3_SiO_5_ to form calcium silicate hydrate (C-S-H gel). The lapping of needle-like Aft crystals with platy-like gypsum crystals formed a stable skeletal structure. C-S-H gel filled the crystal structure, further enhancing the strength of the insulation material. This alteration in microstructure reduced the porosity of the sample and improved the mechanical properties and water resistance. These hydration reactions refine the microstructure of the composite. The intergrowth of ettringite with the plate-like CaSO_4_·2H_2_O crystals forms a more coherent and continuous skeleton, while the C-S-H gel fills residual voids and strengthens the bonding between adjacent crystals. Although microstructural observations are not directly presented in this section, the well-established hydration mechanisms of SAC provide a reasonable interpretation of the mechanical behavior. This refined matrix structure explains the increasing trend in both compressive and flexural strength at the 8% cement dosage. This interpretation is consistent with the peak strength values observed in Figure 10a, which indirectly reflect matrix densification when SAC is added at moderate dosages. However, the addition of excessive cement triggered the expansion of Aft, which destroyed the original stable gypsum crystal structure and altered the stress state inside the gypsum, resulting in cracks inside the gypsum crystal. Therefore, when the cement dosage exceeded 8%, the strength curve showed a downward trend [39].

**Figure 11:**
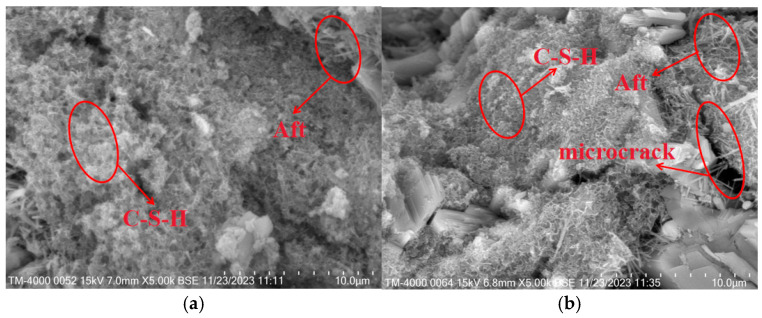
Micrographic comparison of the samples with different cement dosage: (**a**) sample with 8% cement; (**b**) sample with 10% cement.

Figure 10b shows that the apparent density of the sample is positively correlated with the cement dosage. The apparent density of the sample with 10% cement increased from 632 to 663.3 kg/m^3^ with an increase of 4.9%, compared with the reference sample. As the density of cement was greater than that of gypsum, the apparent density of the sample increased after cement replaced some of the gypsum. In addition, a large amount of calcium hydroxide was generated during the initial stage of cement hydration. The increase in Ca(OH)_2_ raised the alkalinity of the pore solution and supplied Ca^2+^, which can activate the latent hydraulic components of the S95 mineral powder. Under these alkaline conditions, the amorphous aluminosilicate phases within the mineral powder partially dissolve and release reactive silicate species, which then react with Ca^2+^ to form secondary C-S-H gel. The silicon dioxide particles, the needle-like Aft crystal, and the calcium silicate dioxide jointly filled the pores of the gypsum crystal, resulting in a more compact interior of the matrix. As shown in Figure 10c, with the increase in cement dosage, the water absorption rate curve initially decreases and then increases, while the softening coefficient curve initially increases and then decreases. The water absorption rate of the sample with the 8% cement was 0.47, which was 11.2% lower than that of the sample without cement. The softening coefficient of the sample with the 8% cement increased from 0.43 to 0.53 with an increase of 21.90%, compared with the reference sample. The incorporation of an appropriate amount of cement optimized the microstructure of the insulation material, strengthening the interaction force between aggregates (Figure 11a). However, when excessive cement was added, many microcracks appeared in the matrix due to the volume expansion of Aft in contact with water. These cracks not only compromised the overall strength of the material but also provided additional pathways for water penetration, so the water absorption rate was increased and the softening coefficient was decreased (Figure 11b).

#### 3.2.2. Effect of Basalt Fiber Dosage on the Material Properties

**Figure 12:**
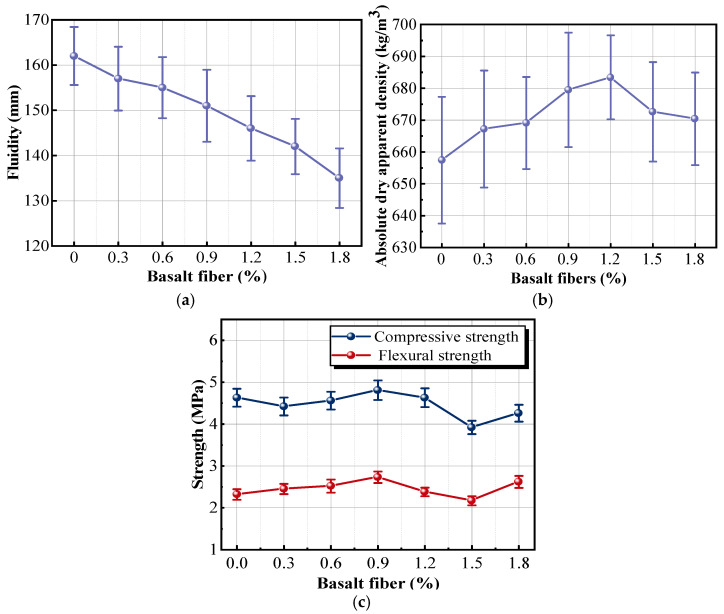
Effects of different basalt fiber dosages on the properties of samples: (**a**) fluidity; (**b**) absolute dry apparent density; (**c**) strength.

The fluidity curve of the insulation mortar shows a linear downward trend, as shown in Figure 12a. The mortar with 1.8% fibers had the poorest fluidity, being 20% lower than that of the mortar without fibers. The results were mainly attributed to the inherent surface tackiness of basalt fibers. As the fiber dosage per unit volume continually increased, the fibers became more easily entangled with each other in the mixing process, eventually forming complex network structures. These structures not only strengthened the interactions between the fibers but also hindered the dispersion of raw materials in the matrix, resulting in fiber agglomeration [40]. Insufficient fluidity of the mortar had a negative impact on the workability of the construction materials, including mortar mixing, sample casting, and surface leveling.

It can be observed from Figure 12b that the apparent density curve with respect to fiber dosage shows an increase within the range of 0% to 1.2% fibers, followed by a decrease in the range of 1.2% to 1.8% fibers. When the fiber dosage was 1.2%, the maximum apparent density of the sample reached 683.4 kg/m^3^. The results indicated that incorporating an appropriate amount of fibers into the mortar could fill the pores and improve the apparent density. However, when the dosage exceeded 1.2%, the fibers struggled to distribute uniformly inside the matrix and formed numerous fiber clusters. These clusters occupied spaces that would otherwise be filled by insulation mortar, creating new pores inside the matrix and damaging the original dense structure. As shown in Figure 12c, the peak compressive and flexural strengths of the sample with 0.9% fibers were 4.81 and 2.73 MPa, representing a 4.6% increase in compressive strength and a 17.67% increase in flexural strength over the reference sample. The results indicated that the matrix was well bonded to the basalt fiber, and the effect of fiber bridging inhibited the development of cracks and the expansion caused by the formation of hydration products [41]. Meanwhile, basalt fibers exhibited great toughness and low stiffness. Compared with the compressive strength, the flexural strength improved more significantly [42]. This increase in flexural strength demonstrates that the dispersed basalt fibers bridge microcracks and restrain their propagation, causing the specimen to develop multiple fine cracks instead of a single penetrating crack. The crack-bridging and pull-out effects enhance toughness, delay failure, and improve the post-cracking deformation capacity of the composite. However, when the dosage exceeded 0.9%, the fiber was not only difficult to disperse and transmit stress but also became a weak point of the sample. During the curing process, the insulation mortar underwent drying shrinkage, and many pores were formed at the interface between the aggregate and cementitious material due to the evaporation and hydration reactions of water inside the matrix. Once the fiber dosage exceeds 0.9%, fiber clustering interrupts matrix continuity and introduces additional pores, while the significant increase in viscosity further reduces workability. Therefore, 0.9% is identified as the optimal dosage that balances improved crack resistance, enhanced toughness, mechanical strength, and acceptable construction performance.

**Figure 13:**
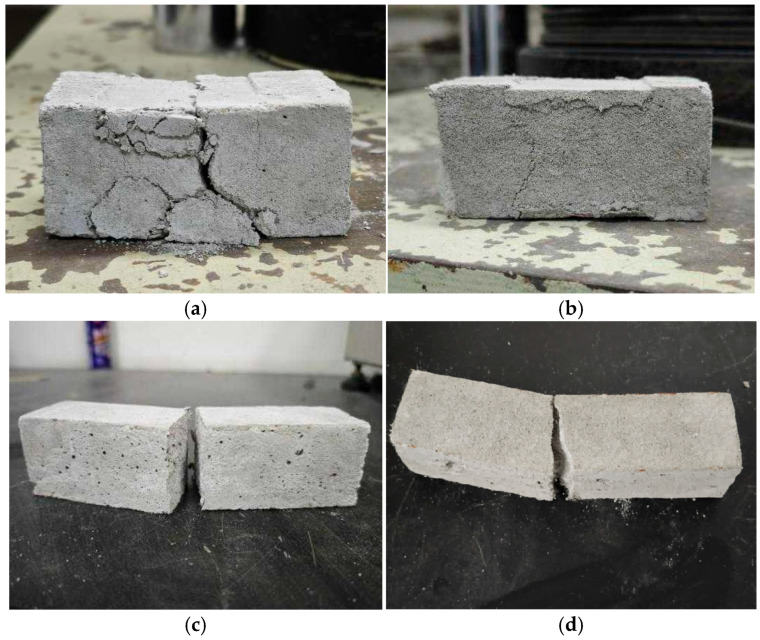
Comparison of samples’ failure patterns: (**a**) compression failure of the sample without fibers; (**b**) compression failure of the sample with 0.9% fibers; (**c**) flexural failure of the sample without fibers; (**d**) flexural failure of the sample with 0.9% fibers.

Figure 13 shows the failure mode of samples subjected to compressive and flexural tests. The reference sample was locally crushed with numerous fragments falling off, and the tensile and shear cracks intersected and penetrated in the middle, showing shear compression failure (Figure 13a). The modified sample exhibited slower crack development, forming microcracks instead of the dominant crack or the falling fragment. This indicated that the fiber effectively played a bridging role to enhance the toughness and alter the failure mode of the sample (Figure 13b). Furthermore, the reference sample rapidly broke with the smooth fracture section after flexural loading, showing brittle failure. Owing to the multiple cracking behaviors of the fiber, the modified sample still maintained the bearing capacity even after damage, thereby delaying failure. The fracture surface was irregular, and many fibers were distributed on both sides of the fracture section (Figure 13c,d).

**Figure 14:**
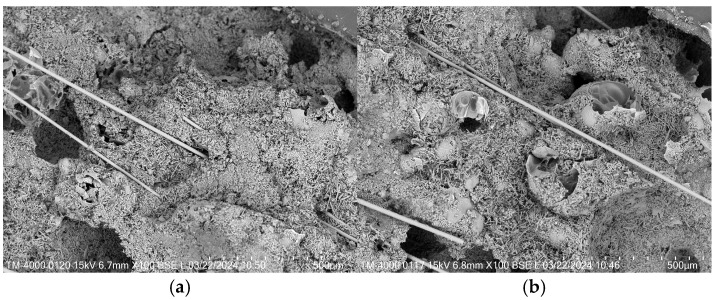
Micrographic comparison of the samples with different fiber dosage: (**a**) sample with 0.9%; (**b**) sample with 1.8% cement.

Figure 14a,b are the SEM images of the sample with 1.2% and 1.8% fibers, respectively. It can be seen that the fiber incorporated into the insulation mortar could fill the internal pores of the matrix, forming a dense structure. This not only delayed the development of cracks but also improved the flexural mechanical property and avoided brittle failure under external load. However, when excessive fibers were mixed into the insulation mortar, the matrix developed numerous pores because the fibers were difficult to disperse and could not be closely connected with the mortar during the mixing process. The structure of the sample became loose, and the apparent density and strength were decreased. In summary, the SEM image analysis was in agreement with the experimental results of the sample.

#### 3.2.3. Effect of Waterproof Agent Dosage on the Material Properties

In this series of experiments, the water resistance of the sample was improved by incorporating paraffin emulsion and spraying SMS. The effect of varying the waterproof agent dosage on the properties of insulation materials was studied.

(1) Paraffin emulsion dosage

**Figure 15:**
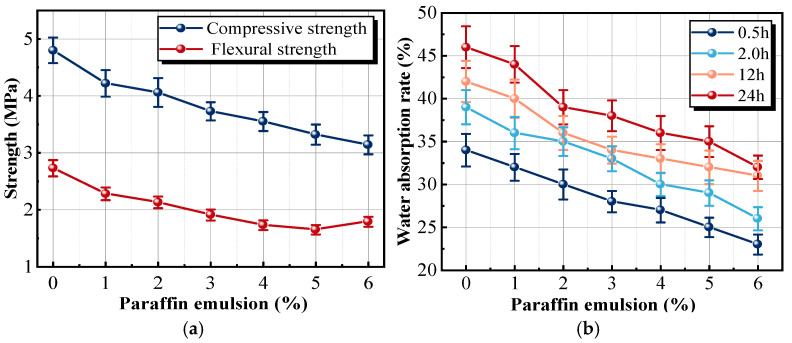
Effects of different paraffin emulsion dosages on the properties of samples: (**a**) strength; (**b**) water absorption rate.

As the paraffin emulsion dosage increased, the strength curves exhibited a linear decline, with a slower rate of decrease beyond 2% dosage, as shown in Figure 15a. The results were attributed to the formation of a waterproof film by the paraffin emulsion in the insulation mortar, which wrapped the surfaces of CaSO4·2H2O crystals and hindered them from lapping through each other [43,44].

Figure 15b shows that the water absorption rate is positively correlated with the emulsion dosage. The water absorption rate of the sample immersed in water for 0.5 h was most significantly affected by the emulsion dosage. When the dosage reached 6.0%, the water absorption rate of the sample immersed in water for 0.5 h was only 23.2%, which was 10.8% lower than that of the reference sample. The surfactant molecule in the paraffin emulsion could directionally arrange on the surface of gypsum particles [45]. Notably, the hydrophilic groups were connected to the surface of gypsum particles, and the hydrophobic groups oriented outwards. The hydrophilic head groups bond to the polar CaSO_4_·2H_2_O crystal surfaces through hydrogen bonding or electrostatic attraction, anchoring the molecules firmly onto the gypsum surface. Meanwhile, the hydrophobic chains extend outward into the pore space. During curing, these oriented molecules gradually merge into a continuous hydrophobic film that encapsulates the gypsum particles, reduces surface wettability, blocks capillary water ingress pathways, and significantly enhances early-stage impermeability. The emulsion formed a dense protective layer after water loss, which could prevent water molecules from entering the interior of gypsum, reduce the erosion of gypsum crystals by water molecules, and improve the waterproof performance of the sample. Furthermore, the particles generated by paraffin emulsion could also be distributed in the insulation mortar, filling the pores and improving the impermeability of the insulation material. However, when the emulsion dosage exceeded 2%, the insulation mortar became too viscous and reduced mutual compatibility, affecting the hydration reaction between the raw material and admixture.

(2) SMS with different dilution multiples

**Figure 16:**
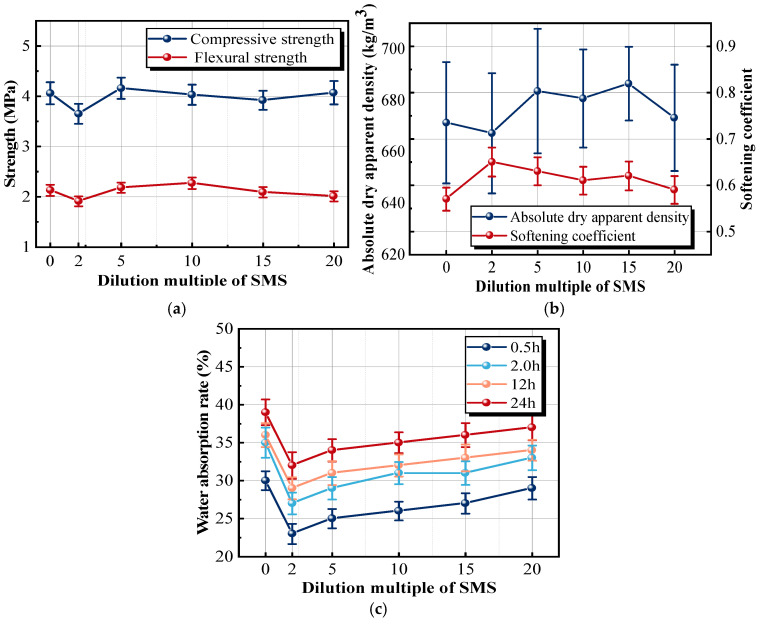
Effects of dilution multiple of SMS on the properties of samples: (**a**) strength; (**b**) absolute dry apparent density and softening coefficient; (**c**) water absorption rate.

To improve the water resistance of the sample, 50 mL of SMS with different dilution multiples should be uniformly sprayed on the surface of the sample. As shown in Figure 16a,b, twice-diluted SMS could reduce the compressive and flexural strengths of the sample, while spraying at other multiples had little effect on the mechanical strength. The SMS reacted with H_2_O and CO_2_ to generate a waterproof film that attached to the surface of the gypsum crystal [46]. Specifically, sodium methyl silicate undergoes hydrolysis to form silanol (Si-OH) groups, which subsequently condense and partially carbonate to produce a cross-linked polysiloxane film. This film tightly adheres to CaSO_4_·2H_2_O crystal surfaces and partially penetrates shallow pores within the matrix, thereby blocking capillary pathways and reducing the surface energy of pore walls. As a result, liquid water infiltration is effectively inhibited, and the impermeability of the material is significantly improved. With the increase in SMS content, the hydration reaction produced a thicker and more continuous polysiloxane film on gypsum crystal surfaces, which excessively coated the nucleation sites and partially obstructed normal crystal growth, reduced the strength, and enhanced the water resistance. When the dilution multiple of SMS was 2 times, the softening coefficient reached the maximum value, which was 14.0% higher than that of the reference sample. The results indicated that the number of surface pores was reduced after surface treatment. SMS could penetrate into the surface layer of the sample, cover the gypsum crystal, or fill the pores between gypsum crystals, and prevent the penetration of water molecules. In addition, when the SMS content increased, the produced film became denser, which improved the softening coefficient.

Figure 16c clearly shows that the water absorption rate of the sample treated with the SMS has decreased to varying degrees, compared with the reference sample. An appropriate concentration of SMS could form a complete film to ensure the formation of a gypsum crystal shape. The methyl group (-CH3) in the film had a waterproof effect, thus reducing the risk of water erosion of the insulation material [46,47]. However, as the immersion time increased, the waterproof films on the surface of the crystal gradually dissolved, and the water absorption rate also increased. After spraying SMS with the dilution of 2, 5, 10, 15, and 20 times, the water absorption rates of the modified samples immersed in water for 0.5h were 21.9%, 23.3%, 24.4%, 25.2%, and 27.4%, which were 38.8%, 30.5%, 24.6%, 20.6%, and 10.9% lower than that of the reference sample, respectively. Actually, the gypsum insulation board was rarely immersed in water for a long time in engineering applications. Considering the manufacturing cost of the board, diluting the SMS by a factor of 5 was enough to meet the water resistance requirement.

In addition, the combined use of paraffin emulsion and SMS provides a complementary waterproofing effect that further enhances the long-term stability of the insulation material. Paraffin emulsion primarily improves internal moisture resistance by forming a hydrophobic coating around CaSO_4_·2H_2_O crystals, thereby reducing early-stage capillary absorption within the matrix. SMS forms a polysiloxane-based surface film that seals surface pores and limits the entry of external water. When applied together, these two mechanisms create a dual protective system composed of internal hydrophobic encapsulation and external pore sealing. This combined action effectively restricts both initial moisture uptake and long-term water penetration, which significantly improves the durability and overall stability of the gypsum-based insulation material.

**Figure 17:**
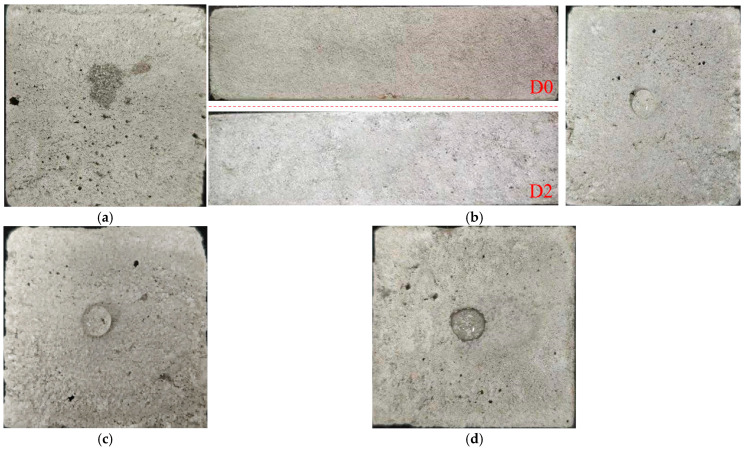
Effect diagram of the samples sprayed with different dilutions of S: (**a**) D0; (**b**) D2; (**c**) D5; (**d**) D10.

To test the water resistance of the samples sprayed with SMS at different dilution multiples, a water droplet was placed on the surface of each sample, and the area of water stain left by the droplet’s penetration was observed. Figure 17 shows that D0, as the reference group, lacked water resistance, and its water droplet quickly immersed the interior. The droplet shapes of D2 and D5 remained full, and the water resistance was the best. However, D2 exhibited severe efflorescence because the dilution multiple of SMS was too small, indicating a high concentration. There were slight wetting marks around the water droplet of D10, indicating that a small amount of water had penetrated into the sample. The water resistance of D10 was inferior to that of D2 and D5. The reason was that the increased dilution multiple reduced the concentration of effective components. Based on the above test results, the optimal dilution multiple of SMS was 5 times.

The compressive strength of the developed insulation material ranged from 3.85 to 4.63 MPa, and the flexural strength ranged from 1.72 to 2.73 MPa. These values are higher than the requirements in the standard GB/T 17669.4-1999 [31], which specifies that the compressive strength shall not be lower than 2.5 MPa and the flexural strength shall not be lower than 1.0 MPa. The measured strengths exceed the minimum limits by approximately 54 to 85% and 72 to 173%. The softening coefficient reached 0.53, which is higher than the lower limit of 0.40 specified in GB/T 50082-2009 [33] for materials used in general indoor environments, giving a safety margin of about 32.5%. The water absorption indicators in GB/T 9775-2008 [34] are short-term measurements and are therefore used only as a reference for evaluating moisture resistance. The dry density of the material is 632.5 kg/m^3^ and meets the usual engineering expectation that lightweight gypsum-based materials should remain below 800 kg/m^3^. These comparisons indicate that the developed insulation material meets the essential performance requirements defined in the relevant standards. At the same time, its performance characteristics show that the material can effectively promote the resource utilization of phosphogypsum and respond to the practical demand for new inorganic insulation materials.

Although SEM was employed in this study to evaluate microstructural damage, the analysis was still primarily based on qualitative visual inspection. To overcome this limitation and enable high-throughput and objective quantification of microstructural and damage features, future research should consider integrating advanced computer vision (CV) techniques. For example, the DeepLab framework [48], which performs semantic image segmentation at the pixel level, can accurately distinguish and quantify the matrix, pores, and crack networks in microstructural images. In addition, the EfficientNet architecture [49], with its compound-scaling strategy and high computational efficiency, is well suited for processing large sets of microstructural images and extracting quantitative descriptors automatically. It should be emphasized that the steady-state thermal conductivity measured on laboratory-scale specimens using the DRE-2C method cannot be directly extrapolated to field-scale thermal performance. Further panel-scale evaluation will be required to substantiate the applicability of the measured values.

## 4. Conclusions

In this paper, a lightweight insulation material utilizing phosphogypsum as its main raw material was studied. The insulation material prepared by the experiment met the standard requirement, not only solving the problem of phosphogypsum accumulation but also satisfying the market demand for new inorganic insulation materials. The main results were summarized as follows:

(1) The study of the initial material ratio indicated that the sample prepared with 1:4 BBR, 1.6 WRB, 0.1% HPMC, and 24% WAE solid content had the best properties. The absolute dry apparent density was 632.5 kg/m^3^, the thermal conductivity was 0.093 W/(m·K), and the compressive and flexural strengths were 3.85 and 1.72 MPa, respectively.
(2) After incorporating SAC in insulation materials, the cement hydration products and gypsum crystals overlapped each other, which could improve the pore structure and mechanical properties. The results showed that the optimal cement dosage was 8%. The compressive strength and softening coefficient of the sample with 8% cement were 4.63 MPa, 2.3 MPa, and 0.53, respectively, which were 20.3%, 35.3%, and 21.90% higher than those of the reference sample.
(3) Basalt fibers effectively enhanced the cracking resistance of the insulation material, but excessive fibers could weaken its workability and strength. The results showed that the optimal fiber dosage was 0.9%, and the fibers could be evenly dispersed in the matrix to form the network structure. The structure not only dispersed the external forces imposed on the sample but also weakened the stress concentration at both ends of the crack in the matrix, which had a positive effect on the stability and toughness. Compared with the reference sample, the flexural strength of the modified sample increased from 2.30 to 2.73 MPa with an increase of 17.67%. Furthermore, the modified sample showed ductile failure.
(4) The hydrophilic and hydrophobic groups of the paraffin emulsion could be oriented on the surface of gypsum particles, forming a dense protective layer after water loss. This not only prevented water molecules from entering gypsum but also avoided the erosion of gypsum crystals. The optimal dosage of the paraffin emulsion was 2.0%. Furthermore, the reaction of SMS with H_2_O and CO_2_ generated a waterproof film, which could cover the gypsum crystal and fill the pores between gypsum crystals, thereby preventing the penetration of water molecules. The film became dense with the increase in the SMS concentration, and the optimal dilution multiples were 5 times. At this time, the modified sample not only met the water resistance requirements but also achieved the optimal level of manufacturing cost and economic efficiency.

## Figures and Tables

**Table 1:** Detailed chemical composition of the raw materials.

Raw Materials	SO_3_	SiO_2_	Fe_2_O_3_	Al_2_O_3_	CaO	MgO	P_2_O_5_	F	Loss on Ignition
Phosphogypsum	4.9	52.3	0.2	0.4	39.3	0.1	1.8	0.6	0.4
Mineral powder	1.9	33.1	/	15.1	39.3	9.96	/	/	0.1
Quicklime	/	0.3	0.1	/	98.0	0.5	/	/	1.1
SAC	17.5	9.8	4.8	19.2	47.5	/	/	/	1.2

**Table 2:** Physical properties of vitrified beads.

Thermal Conductivity W/(m K)	Bulk Density kg/m^3^	Cylinder Compressive Strength MPa	Water Absorption Rate %	Floating Rate %
0.043	85.5	0.115	51.2	78.2

Note: the thermal conductivity was tested in accordance with standard GB/T 10294-2008 [29], and the bulk density, cylinder compressive strength, water absorption rate, and floating rate were determined following the methods specified in standard JC/T 1042-2007 [30] for expanded and vitrified lightweight aggregates.

**Table 3:** Mixing test design group.

Raw Materials	RM	Initial Material Ratio	Modified Material Ratio	Level
Phosphogypsum (g)	/	800	800	/
Mineral powder (g)	/	200	200	/
Quicklime (g)	/	20	20	/
Binder–bead ratio (kg/L)	BRB	1:2~1:8	1:4	1:(1 + ∆) & ∆ = 1
Hydroxypropyl methylcellulose (%)	HPMC	0~0.3	0.1	0.05
Water–binder ratio	WBR	1.5~1.7	1.6	0.1
Waterborne acrylic emulsion solid content (%)	WAE	0~40	24	8
Sulfate aluminate cement (%)	SAC	/	0~10	2
Basalt fiber (%)	/	/	0~1.8	0.3
Paraffin emulsion (%)	/	/	1~6	1
Sodium methyl silicate dilution multiple	SMS	/	2, 5, 10, 15, 20	/

**Table 4:** Physical properties of the modified vitrified beads.

Solid Content %	Bulk Density kg/m^3^	Cylinder Compressive Strength MPa	Water Absorption Rate %	Floating Rate %
0	85.5	0.115	51.5	78.2
8	89.1	0.136	42.5	86.4
16	94.7	0.182	38.2	88.9
24	98.3	0.218	34.2	90.2
32	104.4	0.261	30.7	91.5
40	110.2	0.303	28.6	92.1

## Data Availability

The original contributions presented in the study are included in the article; further inquiries can be directed to the corresponding author.
